# Potential Prognostic Indicators for Patients With Retinal Vein Occlusion

**DOI:** 10.3389/fmed.2022.839082

**Published:** 2022-05-25

**Authors:** Shan Yin, Yanyan Cui, Wanzhen Jiao, Bojun Zhao

**Affiliations:** ^1^The First Clinical Medical College, Shandong University of Traditional Chinese Medicine, Jinan, China; ^2^Department of Ophthalmology, Liaocheng People’s Hospital, Liaocheng, China; ^3^Department of Ophthalmology, Shandong Provincial Hospital Affiliated to Shandong First Medical University, Jinan, China

**Keywords:** branch retinal vein occlusion, central retinal vein occlusion, visual activity, prognostic, indicators

## Abstract

The second most prevalent cause of retinal vascular disease is retinal vein occlusion (RVO). RVO raises intravascular pressure in the capillary and veins, triggering vessel barrier collapse and subsequent leaking of blood or plasma components into the tissue (edema). Macular edema (ME) is a major complication of RVO that results in significant visual impairment. Laser therapy, intravitreal steroid injections, and vascular endothelial growth factor (VEGF) inhibitors are the major therapeutic techniques. Different therapies reduce ME of RVO and improve visual activity. However, some people have no impact on the resolution of ME, while others have a poor visual prognosis despite full ME cure. There are many investigators who studied the relationship between indicators of various instruments with visual activity. However, a summary of those findings is currently lacking. Therefore, we will focus on the predictive factors of different studies associated with positive visual activity outcomes, which would be very useful and important to help address both treatment expectations and methods for patients with RVO.

## Introduction

Retinal vein occlusion (RVO) is one of the common causes of retinal vascular disease (1). Both branch RVO (BRVO) and central RVO (CRVO) are correlated to vision loss and reduction in vision-related quality of life (2, 3). Although RVO is more common in elderly people, it can occur in younger people as well (4), possibly affecting their ability to live and work. The use of anti-vascular endothelial growth factor (VEGF) therapeutics to treat macular edema (ME) caused by RVO is a significant step forward in improving visual outcomes in individuals with BRVO and CRVO (5, 6). However, successful treatment is often temporary. Following several intravitreal injections of anti-VEGF, some individuals have no impact on the resolution of ME, while others have a poor visual prognosis despite full resolution of the ME. Therefore, this summary will focus on the predictive factors associated with positive visual activity (VA) outcomes, which would be very useful and important to help address both treatment expectations and approaches for patients with RVO.

### Gender

The HORIZON extension study reported the association of men with good final vision in patients with BRVO (7). The normal range for hematocrit in this research was 40.7 to 50.3% for male patients and 36.1 to 44.3% for female patients. A stronger oxygen-carrying capacity in men may avoid ischemia and consequent high levels of VEGF and its related problems, which may explain why men have better visual results and responses to therapy. Similarly, in a cross-sectional countrywide survey of 557 naive individuals with RVO in Korea, female patients were considerably more likely to have lower vision than male patients with BRVO (8). Additional research may be required to determine if other sex-related characteristics (e.g., hormone levels, lifestyle, behaviors, and environmental factors) influence the results of VEGF suppression in retinal vascular disorders.

### Age

Younger age was related with not only a 15-letter improvement in BCVA, but also a good final best-corrected visual acuity (BCVA) in patients with CRVO (7). Chatziralli and Winegarner also stated that final visual acuity is negatively related to age (9, 10). There are many possible reasons why age may contribute to poor efficacy of anti-VEGF drug therapy. Goff and Bishop (11) discovered biochemical alterations in the vitreoretinal interface as well as the thickening of the inner limiting membrane (ILM) with age, both of which potentially impede anti-VEGF agent diffusion into the retinal layers. Another study found that the vitreous body undergoes irreversible changes as it ages, including changes in the collagen fibrils and hyaluronic acid components, condensation of the vitreous gel, and the creation of optically empty spaces (12). Previous research has also revealed that in vitrectomized eyes, the clearance rates of pharmaceutical compounds from the vitreous were quicker, and as a result, the efficacy of anti-VEGF drugs was reduced (13, 14). Given these findings, alterations in the vitreous structures related to aging may impact the solubility and diffusion rates of anti-VEGF drugs, as well as increase the rate of aflibercept elimination in elderly patients. Finally, retinal circulation could change with age, especially when the number of retinal capillaries decreases and arteriosclerotic changes in retinal arteries develop (15). The reduction in retinal blood flow may hasten the release of VEGF. As a result, in aged persons, a decline in anti-VEGF response may be associated with a reduced retinal flow and elevated VEGF levels. At the same time, the retinal pigment epithelium (RPE) layer may alter with age, such as growing more pleomorphic, accumulating lipofuscin, and experiencing a decrease in cell quantity (15). Since RPE layer activities are critical to photoreceptor cell health, disruption of these processes may affect the responses to aflibercept therapy in older individuals with BRVO.

### Smoking

To date, only Sophie and associates reported that never smoked was related not only with a 15-letter improvement in BCVA but also with a good final BCVA in patients with CRVO (7). No such correlation was found in patients with BRVO. Smoking inhibits the endothelial function, promotes inflammation and lipid modification, disrupts the balance of antithrombotic and prothrombotic factors, and accelerates the atherothrombotic events in the body (16). Another well-known impact of smoking is that it increases the number of circulating white blood cells and the aggregation of white blood cells (17). Smoking has been demonstrated to reduce retinal blood flow and the capacity of retinal vessels to autoregulate in response to hyperoxia (18–21). As a result, increasing age and smoking worsen the vascular disease, which lowers the chance of favorable visual outcomes in individuals with CRVO.

## Blood-Related Indicators

### Red Blood Cell Distribution Width

Red blood cell distribution width (RDW), a metric that assesses variation in red blood cell size, is an indication of erythrocyte heterogeneity (22). For the first time, the study of Ozkok et al. (23) showed a link between RDW value and vision results in patients with RVO. RDW values were found to be considerably greater in patients with RVO compared to an age-matched control group, and higher RDW values were related to a worse beginning vision and a lower final vision. Similarly, Pinna et al. (24) observed that patients with both RVO and BRVO had significantly higher RDW values. In a univariate logistic regression analysis, RVO was significantly associated with RDW. However, a multivariate logistic regression analysis found no statistically significant relationship between RVO and RDW. The relationship between vision and RDW needs to be verified by further studies. The mechanism behind the link between RDW and clinical outcomes in patients with RVO remains unknown, while oxidative stress (25) and chronic inflammation, as well as endothelial dysfunction, might be involved (26).

To sum up, in patients with RVO, RDW value may be a predictive indicator for vision. This affordable and widely accessible biomarker may be useful as a prognostic factor. More research with bigger sample numbers and prospective methods is recommended to acquire more conclusive results.

### Mean Platelet Volume

Mean platelet volume (MPV), a key indicator of platelet manufacturing rate and size, has been correlated to platelet activation. Onder et al. (27) and Bawankar et al. (28) found that MPV was considerably greater in hypertensive individuals with BRVO and CRVO when compared to the hypertension control group. Furthermore, Sahin et al. (29) discovered that MPV levels were considerably higher in patients with RVO in a retrospective analysis of 193 patients with RVO and 83 healthy control persons. Yilmaz et al. discovered a similar effect in another retrospective analysis of 83 patients with RVO and 85 controls (30). Pinna et al. (24) discovered significantly higher median values of MPV in patients with RVO, BRVO, or CRVO compared with their respective controls. MPV was responsible for 30% of the attributable risk for RVO.

Previous research has revealed that systemic and local inflammation may play a role in the development of RVO by causing atherosclerosis and hypercoagulability. Changes in MPV and RDW represent the severity of inflammation in a variety of disorders because systemic inflammation not only affects the survival of platelets and red blood cells, but also deforms their membranes (31). Based on the above findings, we speculate that MVP may also be a predictor of final visual acuity. However, there are no studies on the relationship between MVP and visual acuity in patients with RVO. More prospective studies on MPV as a predictive biomarker in patients with RVO are required to determine the pathophysiologic and clinical relevance of elevated MPV in patients with RVO.

### Hematocrit

The HORIZON extension research (7) discovered a negative association between low hematocrit and ultimate vision in patients with BRVO. This might be explained by the lower oxygen-carrying capacity of blood in patients with low hematocrit, which could increase retinal hypoxia and induce higher activation of hypoxia-regulated genes, such as VEGF, as well as perhaps worsen ischemia damage to macular cones in some patients. The significance of the discovery is underlined by the observation that normal vs. low hematocrit was similarly linked with a 15-letter improvement in BCVA in individuals with BRVO. In contrast to BRVO, normal vs. low hematocrit was not connected with favorable visual results in CRVO, which might simply be due to chance, but it could also be due to specific traits that CRVO eyes do not share with BRVO eyes. At present, the HORIZON extension study descried above is the only one looking at the link between RVO patients’ hematocrit and visual acuity.

## Systemic

The HORIZON extension study (7) found a history of hypertension and lower mean diastolic ocular perfusion pressure (OPP) associated with 15-letter BCVA improvement in patients with CRVO. However, these indicators were not associated with good final visual acuity. High systolic blood pressure raises systolic OPP, which compensates for higher resistance, slow flow, and reduced autoregulation to prevent retinal ischemia (32). The relationship between higher systolic OPP and ultimate CST ≤ 250 μm shows that increased systolic perfusion pressure may be beneficial in CRVO. On the other hand, persistent high ocular perfusion throughout the cardiac cycle may damage retinal capillaries because decreased diastolic perfusion pressure was related to a 15-letter improvement in BCVA in patients with CRVO. HORIZON extension study found no correlation between hypertension and 12-month BCVA in patients with BRVO. According to the study by Asano et al., 6-month post-IVR BCVA was positively associated with pre-IVR systolic blood pressure (SBP). Furthermore, a multiple regression analysis revealed that pre-IVR SBP was an independent predictor of 6-month post-IVR BCVA (33). Their findings may not be representative because of the small number of patients enrolled. Nevertheless, further studies are needed to verify the relationship between hypertension and visual acuity in patients with RVO and to better understand the effects of systolic and diastolic OPP on normal and diseased retinal vessels.

## Ocular Factors

### Baseline Best-Corrected Visual Acuity

In both BRVO and CRVO, there was a high association between good BCVA at the outset of therapy and good final BCVA (7, 10, 34). Consistently, the final VA at 12 or 24 months was lower in patients with poor baseline VA in the BRAVO, BRIGHTER, and LUMINOUS investigations, highlighting the necessity for early treatment initiation (35–37). In the therapies of retinal and choroidal vascular diseases, the association between good starting vision and good final vision outcome is likely because good starting vision reflects less severe disease and, as a result, a lower likelihood of irreversible damage to photoreceptors or other retinal cells.

The baseline BCVA is an important predictor of VA gains with treatment (34, 38). In the HORIZON extension study, poor initial BCVA is related to considerable improvements in BCVA of patients with CRVO (7). Patients with poor BCVA have a longer path to good final vision, but that longer path affords more opportunities for development. Surprisingly, there was no correlation between poor beginning BCVA and improvement in BCVA of 15 letters in patients with BRVO (7). However, patients with BRVO with lower baseline VA exhibited statistically larger VA improvements at 12 and 24 months, according to the BRAVO and BRIGHTER trials (35, 37). In keeping with these findings, quantitatively larger VA increases were reported among the LUMINOUS research in patients with BRVO with baseline VA of 39– < 60 letters (36). Patients with a baseline VA of 60– < 74, on the other hand, maintained their VA at 12 months with appropriate ranibizumab therapy.

### Injection Frequency

In the LUMINOUS research, higher injection frequency was related to higher VA increases in the worldwide population across all enrolling nations (36). The good mean letter gains shown in Year 1 imply that patients with BRVO may not require as many ranibizumab injections as patients with other retinal disorders (such as DME and nAMD) to attain satisfactory visual results (36, 39, 40). Nevertheless, it does confirm the need of maintaining a proper dose to get excellent BCVA results. However, Callizo and colleagues demonstrated that the number of injections had no effect on the change in BCVA in patients with RVO (41), probably because of different kinds of patients with RVO included in the two studies. The relationship between injection frequency and visual acuity in patients with CRVO needs to be further studied.

### Duration

The BRIGHTER study discovered that illness duration influenced the treatment results (37). Patients with a BRVO duration of < 3 months and 3–months had statistically better BCVA outcomes than those with a longer illness duration (6–9 months, 9–12 months, and > 12 months). Similarly, Chatziralli et al. concluded that the duration of RVO ≥ 3 months was substantially related to poorer visual acuity in patients with BRVO (9). These results demonstrate the importance of early treatment for patients with BRVO.

## Fundus Photographs

On fundus photos, the fovea was determined to be an area of around 1 disc diameter in size, located in the area of deepest xanthophyll pigmentation, roughly 2.5 disc radii temporal, and somewhat inferior to the center of the optic disc (42). Powers et al. (43) studied the relationship between fovea-involving intraretinal hemorrhage (IRH) and visual acuity of acute, treatment-naive BRVO. They showed that despite receiving more intravitreal anti-VEGF injections, eyes with foveal hemorrhage at presentation had worse visual results than those without fovea involving IRH. In the univariate analysis, fovea involving IRH was found to be a significant predictor of final visual result; however, this significance was lost in the multivariate analysis. The difference might be related to the study’s small sample size, as well as other unknown confounding variables. Future research is needed to determine if fovea-involved IRH is a reliable predictor of eventual visual result in eyes with BRVO.

## Ultra-Widefield Fluorescein Angiography

Ultra-widefield fluorescein angiography (UWF-FA) accurately assesses leakage, ischemia, and non-perfusion in both the macula and the retinal periphery (44, 45). Several cross-sectional investigations have discovered that retinal non-perfusion and its correlate, the ischemia index (ISI), are inversely related to visual acuity (44, 46). Baseline retinal ischemia indicated by UWF-FA is substantially related to disorganization of retinal inner layers, which predict poor visual acuity at follow-up (47). At present, there is no literature showing the direct relationship between UWF-FA related indicators and final visual acuity.

Previous research has shown that the ISI may have both predictive and prognostic significance; however, accurately assessing the non-perfusion area and total area of the visible retina to compute the ISI is a significant difficulty. Although the ISI is an imperfect measure of non-perfused retina, recent advances in UWF-FA software that reduce the amount of peripheral distortion inherent in widefield images have resulted in more precise calculations of percentages of peripheral non-perfusion, which may help characterize disease severity (48). Other topographic non-perfusion measurement approaches, such as concentric rings centered on the fovea, have also been examined (49, 50).

## Electroretinography

Electroretinography (ERG) is a technique for objectively assessing the physiological health of the retina, and it has been widely used in various eye diseases. Full-field ERGs (ffERG) can assess the retinal function of the whole retina (51), and Miyake et al. developed the focal macular ERGs (fmERGs) to assess macular function in various retinal disorders (52). The assessment of prognostic visual acuity in patients with RVO by ERG is summarized below, but the number of patients enrolled in each study was relatively small.

Ogino and colleagues used fmERG to evaluate the physiology of the macula in patients with CRVO and showed that the baseline relative amplitudes of the a-wave, b-wave, and photopic negative response (PhNR) were negatively correlated with baseline LogMAR BCVA (53). Nishimura et al. (54) reported that the baseline amplitudes of the a- and b-waves and the Σoscillatory potentials (ΣOPs, the amplitudes of the OP1, OP2, and OP3) of the fmERG were significantly correlated with the BCVA at month 12, and no correlation between baseline PhNR and 12-month BCVA was observed. However, Moon et al. indicted that pretreatment PhNR relative amplitude showed a good predictive value for good visual outcome at 22 weeks after treatment (55). Therefore, we speculate that PhNR can predict short-term visual acuity in patients with CRVO but has no predictive effect on long-term visual acuity. In a study by Nishimura et al. (56) the average amplitudes of all components, except the a-wave of the fmERGs, were attenuated in the affected eyes at the baseline, and the fmERG amplitudes were not correlated with the final BCVA in patients with BRVO.

Nishimura et al. also reported the reduced amplitudes of the ffERG, ΣOPs, and the PhNR remained unchanged for the 12-month period in patients with CRVO, but they did not state the relationship between amplitudes of the ffERG and BCVA (54). Through their research, they concluded that the recovery of the whole retinal function was minimal in contrast to the recovery of the macular function. Thus, fmERG may be a better predictor of visual acuity than ffERG.

Multifocal ERG (mfERG), which shows the functional effects of the inner retinal layers between the retinal vascular arcades (57), has been used to indicate RVO-related retinal dysfunction (58). Many researchers agree that mfERG is aberrant in patients with RVO (59, 60). Only Bulut et al. reported that no correlation was detected between BCVA and mfERG P wave amplitudes or implicit times (61).

## Microperimetry

Microperimetry (MP) is a method for assessing visual function that focuses on the macula and employs targeted light stimulation to quantify contrast sensitivity in specific macular areas. The test provides precise maps of retinal sensitivity (RS) that represent the visual function of the whole macula by employing both static and dynamic stimuli. While the BCVA has long been the most common method of assessing visual function, it focuses primarily on the central foveal function. Microperimetry evaluation of RS and fixation can examine a larger parafoveal region and connect with other complicated binocular processes like reading (62).

According to one research, retinal sensitivity evaluated with MAIA (Center Vue, Padua, Italy) microperimetry 1 day after anti-VEGF therapy was positively associated with BCVA at 1 month in eyes with BVOME (63). Fujino et al. discovered that changes in retinal sensitivity at 1 month were negatively associated with LogMAR BCVA at 3 months after anti-VEGF therapy, showing that retinal sensitivity can be used to predict visual acuity (64). This suggested the potential usefulness of retinal sensitivity in predicting the BCVA prognosis.

## Optical Coherence Tomography

Spectral-domain optical coherence tomography (SD-OCT), a non-invasive optical imaging method, is the standard and the most often used technology for objectively evaluating and monitoring therapy efficacy by assessing ME resolution. SD-OCT can analyze more information as the resolution increases. OCT predictors may give predictive value for visual outcomes and visual gains following anti-VEGF drug injection in treatment-naive RVO-ME using OCT analysis based on the identification of specific quantitative or qualitative criteria.

### Central Subfield Thickness

The large studies, such as *post hoc* analyses of standard care vs. corticosteroid for RVO 1 (SCORE1) and the study of comparative treatments for RVO 2 (SCORE2) clinical trials, found no link between baseline central subfield thickness and visual outcomes in RVO eyes treated with grid laser photocoagulation, anti-VEGF therapy, or intravitreal triamcinolone (65, 66). Similarly, a retrospective investigation of 208 BRVO and 123 CRVO eyes treated with anti-VEGF, intravitreal triamcinolone, laser, or a combination according to physician judgment found no link between baseline central subfield thickness and VA at 6 months in either group (38). Chan et al. also discovered that baseline subfield thickness did not predict visual results, but that greater macular thickness variations were linked to worse VA outcomes (67). The effect of mechanical stress on retinal cells might be one reason for the association between macular thickness changes and impaired macular function.

### Choroid

The choroid is a highly vascularized network of tissue that supplies oxygen to the outer third of the retina. Enhanced depth imaging optical coherence tomography (EDI-OCT) has made it possible to investigate the choroid at a greater depth, especially by assessing choroidal thickness. Rayess et al. showed that a higher initial subfoveal choroidal thickness was a positive predictor of favorable visual outcomes in patients with BRVO by univariate analysis but not multivariate analysis (68). In contrast Yu et al. (69) reported that choroidal thickness did not impact VA at any time point in patients with BRVO. However, another study examined in detail the choroidal change of RVO (70). In this study, EDI-OCT images were binarized using ImageJ, and the stromal area to total choroidal area (S/C) ratio was computed by measuring subfoveal cross sectional areas of the luminal, stromal, and total choroid across a 1500-m span using EDI-OCT. They discovered that baseline central choroidal thickness was unrelated to baseline or improvement in VA. At 1, 3, and 6 months, the baseline S/C ratio was substantially linked with VA improvement. The total choroidal area and stromal area were not shown to be substantially linked with clinical outcomes. This demonstrated that the increase in choroidal thickness was mostly attributable to stromal area growth rather than luminal region expansion, and that this stromal region expansion indicated choroidal stromal edema. The S/C ratio is a better predictor of treatment response than choroidal thickness, highlighting the need of looking at choroidal structure rather than gross appearance.

### External Limiting Membrane

External limiting membrane (ELM), which is regarded as the zonula, adheres between photoreceptors and Müller cells, appears to be a characteristic of photoreceptor function, and its condition may directly indicate the capacity for visual function and/or recovery. Yiu et al. (71) reported that in univariate analyses and multivariate models, baseline ELM disruption was associated with vision before treatment and not with visual acuity at 6 months post-treatment in patients with RVO. In a multivariate analysis, Tang et al. (72) found that the baseline extent of ELM disruption was strongly linked with BCVA at baseline, 1 month, and 3 months following anti-VEGF therapy in patients with RVO. At baseline and 3 months, there was a 0.04 logMAR (2 letters) and 0.02 logMAR (1 letter) loss of vision for every 100 μm of further ELM disturbance. Similarly, several studies have found that having an intact initial ELM predicts a better visual result after anti-VEGF medication in RVO (69, 73). In summary, ELM is an important indicator for predicting final visual acuity in patients with BRVO.

### Ellipsoid Zone Disruption

In the bivariate analysis, Tang et al. (72) found that baseline ellipsoid zone (EZ) disruption was negatively connected with BCVA at baseline, 1 month, and 3 months, but not in the multivariate analysis. At baseline, the degree of ELM disruption was shown to be highly associated with the degree of EZ disruption. The level of EZ disruption was not reflected as an independent visual acuity predictor in the multivariate analysis, which might explain why the status of ELM could represent the EZ integrity to some extent. Tang et al. also found that the magnitude of EZ disruption at baseline and its changes over time were related to BCVA improvement at 3 months in the multivariate analysis. At the 3-months treatment, there was a 0.03 logMAR (1.5 letters) improvement in BCVA for every 100 μm of extra increase at the baseline. According to Chan et al. (74), the first 3 months of EZ integrity are reliable predictors of 1-year VA. Over the course of 3 months, a recovery of around 100 μm of EZ disruption predicts a one-line VA increase over 1 year. They speculated there seems to be a 3-month window in which retinal structural evolution influences future VA.

### Hyperreflective Foci

On SD-OCT, hyperreflective foci (HF) were characterized as tiny discrete, well-circumscribed, dot-shaped lesions with reflectivity equal to or greater than the RPE band (75). In a multivariate study, Tang et al. (72) observed that HF > 20 at baseline was related to poorer BCVA at each time point. However, no significant relationships with HF at baseline or its changes over time and BCVA improvement at 3 months were detected. Yiu et al. (71) reported that in univariate analyses, not in multivariate regression, the increases in BCVA between baseline and month 7 were linked with intraretinal HF presence. In ischemic CRVO and BRVO, the quantity of HF in the outer retinal layers was linked to a poor visual result according to Bin Mo et al. All their studies pointed to HF as a biomarker for poor visual outcomes. Although the exact cause of HF is unknown, recent research has suggested that it may be caused by extravasated lipoproteins (the precursors of hard exudates), lipid-laden macrophages, photoreceptor degeneration, and activated or over-phagocytosed RPE cells or RPE metaplasia and microglia in an inflammatory environment (75–77). HF appears to reflect fluid extravasation produced by the breakdown of the blood–retina barrier in RVO, which eventually leads to hard exudates and ME.

### Disorganization of the Retinal Inner Layers

Disorganization of the retinal inner layers (DRIL) was defined as the horizontal extent of the disarray of the boundaries between the ganglion cell–inner plexiform layer complex, inner nuclear layer, and outer plexiform layer (78). A total of 136 eyes in patients with RVO were included in one study (79), and followed up after 8 months. They showed that baseline DRIL correlated with baseline BCVA. The results are entirely consistent with the findings of Babiuch et al. (80), Chan et al. (74). And Mimouni et al. (79) reported that for every 100 um of DRIL at baseline there was 0.03 logMAR less improvement in BCVA at 8 months. And reduction in DRIL at 4 months and 8 months correlated with good BCVA outcome at 8 months. Chan et al. reported that initial 3-month evolution of DRIL were robust predictors of 1-year VA (74). Recovery of approximately 190 mm of DRIL over 3 months predicted a 1-line VA gain over 1 year. Babiuch et al. (80) also adjusted for baseline VA and found that in the CRVO/HRVO group, the absence of DRIL at baseline was associated with larger VA increases at 6 months. At 6 and 12 months, increasing DRIL scores in CRVO/HRVO was linked with less VA improvement. DRIL length at baseline was a predictor of poorer VA at 12 months, according to Yu et al. (69), and DRIL length at 12 months was a predictor of worse final VA.

The extent of DRIL was manually measured and, from a practical perspective, it may be difficult to measure the extent of DRIL. Automatic and objective measures with automated software may simplify this process in the future, providing quantifiable measurements to the practicing ophthalmologist. This would allow the physician to efficiently employ the findings of this study routinely.

## Optical Coherence Tomography Angiography

Optical coherence tomography angiography (OCTA) is a non-invasive approach for studying retinal microvasculature. Without dye injection, OCTA depicts the eye microvasculature and its three-dimensional structure in amazing detail and excellent resolution. This enables a more thorough view of the superficial and deep plexuses, allowing for the assessment of vessel density and vasculature in different retinal layers (81). As a result, OCTA has helped researchers better understand the association between layer-specific retinal microvascular alterations and visual outcomes in patients with RVO.

### Macular

At baseline, the BCVA was negatively associated with the choriocapillaris flow area, the whole en face (3 mm × 3 mm) vessel density (VD), and the overall parafoveal VD in both the super capillary plexus (SCP) and deep capillary plexus (DCP) in the BRVO, but not in CRVO (82, 83). A cross-sectional study combining OCT and OCTA images showed a significant negative correlation between logMAR VA and FAVD/DFD [the fovea–AV crossing distance (FAVD), optic disc–fovea distance (DFD)] values, but no correlation between logMAR VA and the DAVD/DFD [optic disc-AV crossing distance (DAVD)] in patients with BRVO (84).

The 1-month posttreatment logMAR BCVA was negatively associated with the choriocapillaris flow area in BRVO but not CRVO (82, 83). One study (85) showed that in CRVO following ME resolution, the logMAR BCVA after intravitreal aflibercept treatment was negatively connected with retinal perfusion area for both the SCP and the DCP. In addition, FAZ area in both SCP and DCP following intravitreal aflibercept therapy was positively connected with posttreatment logMAR BCVA. But the other study did not show any significant correlation of VA with FAZ and VD in BRVO after resolution of the CME (86). Winegarner et al. (10) showed that BCVA at 12 months after treatment positively correlated with the initial VD in the SCP and DCP. VD and FAZ were not altered considerably over a year. A multivariate regression analysis found that visual results 12 months following anti-VEGF medication were related to better VD in the SCP and DCP, as well as a smaller FAZ in the SCP and DCP.

Vessel density in the DCP was the most important OCTA metric that was highly connected with BCVA. Studies on RVO animal models revealed that the SCP had more perfusion than DCP because it is directly related to the retinal arterioles. On the other hand, DCP is formed predominantly by venous collecting channels and may be more prone to venous blockage (87, 88). Furthermore, the vascular anatomy of the DCP that involves direct connectivity with major veins, as well as the lack of vascular smooth muscles make DCP most prone to hemodynamic disturbances following RVO and the resulting hypoperfusion than the SCP (89–91). The critical role of the DCP in the nourishment of the watershed zone, which is positioned between the inner nuclear layer (INL) and the outer plexiform layer (OPL), includes neuronal synapses transferring vision signals from photoreceptors, therefore hypoperfusion of the DCP might be deleterious to visual function (92).

The cause for these inconsistencies in anatomical results, in contrast to considerable functional gain by VD in RVO, is unknown; however, various possibilities have been suggested. A consistent limitation of these investigations is the image quality acquisition when ME is present. Due to the possible signal attenuation and difficulties in exact retinal plexus delineation of the automatic segmentation tool, severe ME interferes with correct interpretations of OCTA parameters. Furthermore, microvascular macular alterations may cause OCTA quantitative results to be distorted. When there is more ME, vascular density may be exaggerated owing to vessel dilatation. If the blood flow of the dilated vessel is less than 0.3 mm/s on OCTA (90), it is viewed as a cystoid space rather than a vascular venous engorgement.

### Disc

In Nicolai’s cohort (93), in CRVO eyes inside disc VD differences between baseline and 4-months post-operative group were statistically significant. The difference in peripapillary VD between the baseline and 1-month groups, as well as the baseline and 4-month groups, was statistically significant. In the fellow eyes, differences in VD values at baseline and posttreatment were not statistically significant. At baseline, 1 month, and 4 months posttreatment, variations in whole image, inside disc, and peripapillary VD were highly statistically significant between the affected eye and the fellow eye. There is limited literature on optic disc blood flow. At present, we have not found literature on the relationship between visual acuity and optic disc blood flow density.

## Laser Speckle Flowgraphy

Laser speckle flowgraphy (LSFG) quantifies circulation in the optic nerve head (ONH), choroid, and retinal arteries *in vivo* by utilizing the laser speckle phenomenon (94, 95). The main output of LSFG is mean blur rate (MBR), a quantitative measure of erythrocyte movement based on laser speckle patterns, and most studies use MBR or a ratio of MBR as a direct correlate of blood flow. Toshifumi Asano et al. used LSFG to assess vessel MBR, tissue MBR, and overall MBR separately in the ONH of patients with BRVO. Their results showed that pre-IVR, 1-month, 6-month post-IVR, overall MBR, and vessel MBR were all negatively correlated with 6-month post-IVR logMAR BCVA. Furthermore, a multiple regression analysis revealed that pre-IVR overall MBR was the independent prognostic factor of 6-month post-IVR logMAR BCVA (33). Matsumoto et al. (96) measured the MBR of the ONH in patients with CRVO to assess microcirculation at the ONH. Both before and after treatment, there was a significant negative correlation between logMAR visual acuity and MBR, with the correlation becoming stronger after treatment. These findings imply that measurements of the independent factors MBR can be used to assess logMAR visual acuity in patients with BRVO and CRVO.

## Conclusion

The biomarkers that predict future BCVA in patients with RVO may significantly enhance risk assessment and management decisions. We have summarized published potential prognostic indicators for patients with RVO. The reliable prognostic indicators have been summarized in Tables 1, 2. The current researches on the relationship between UWF-FA parameters and visual acuity in RVO patients are cross-sectional studies, and longitudinal studies are lacking. In addition, there are few studies on the relationship between MP and ERG parameters and prognostic visual acuity in patients with RVO, and the sample sizes of these studies are small. Future studies can focus on the above aspects, and there will be a trend toward multimodal imaging to assess the prognosis of patients with RVO.

**TABLE 1:** The relationship between non-imaging indicators and visual acuity in patients with retinal vein occlusion.

Non-imaging parameters	Relationship with visual activity
Gender	Men have better final BCVA in BRVO patients
Age	Younger patients have better final BCVA
Smoking	Never smoking was related with good final BCVA in CRVO patients
Red blood cell distribution width	Higher RDW values were related with lower final vision
Hematocrit	Low hematocrits were related with lower final vision
Hypertension	History of hypertension and lower mean diastolic OPP associated with BCVA improvement in CRVO patients
Baseline BCVA	Good baseline BCVA were related with good final vision.
Duration	Duration of < 3 months had statistically better BCVA outcomes

*BRVO, branch retinal vein occlusion; BCVA, best-corrected visual acuity; OPP, ocular perfusion pressure.*

**TABLE 2:** The relationship between imaging indicators and visual acuity in patients with retinal vein occlusion.

Imaging parameters	Relationship with visual activity
Fundus photographs	Fovea involving IRH was related with lower final VA
Focal macular ERGs	Pre-treatment PhNR relative amplitude was inversely correlated with LogMAR BCVA
Optical coherence tomography angiography	Initial VD in the SCP and DCP was negatively connected with final BCVA
Laser speckle flowgraphy	There was a significant negative correlation between logMAR visual acuity and MBR
Optical coherence tomography	
Choroidal thickness	The baseline S/C ratio was substantially linked with VA improvement.
External limiting membrane	Intact initial external limiting membrane predicts a better final VA
Ellipsoid zone disruption	Baseline ellipsoid zone disruption was negatively connected with BCVA
Hyperreflective foci	Hyperreflective foci > 20 at baseline was related with poorer BCVA
Disorganization of the retinal inner layers	Baseline DRIL presence was related with VA improvement.

*IRH, intraretinal hemorrhage; VA, visual acuity; PhNR, photopic negative response; VD, vessel density; SCP, super capillary plexus; DCP, deep capillary plexus; MBR, mean blur rate; S/C ratio, the stromal area to total choroidal area ratio.*

## Author Contributions

SY wrote the manuscript. All authors contributed to manuscript revision, read, and approved the submitted version.

## Conflict of Interest

The authors declare that the research was conducted in the absence of any commercial or financial relationships that could be construed as a potential conflict of interest.

## Publisher’s Note

All claims expressed in this article are solely those of the authors and do not necessarily represent those of their affiliated organizations, or those of the publisher, the editors and the reviewers. Any product that may be evaluated in this article, or claim that may be made by its manufacturer, is not guaranteed or endorsed by the publisher.
